# Antifungal use with and without fungal diagnoses among patients with septic shock in a retrospective multicenter cohort

**DOI:** 10.62675/2965-2774.20260094

**Published:** 2026-08-25

**Authors:** Robert J. Flick, Li Yan, Nicholas A. Bosch, Anica C. Law, Khyzer B. Aziz, Chad H. Hochberg, Joseph Levy, Theodore J. Iwashyna

**Affiliations:** 1 Johns Hopkins University School of Medicine Division of Pulmonary and Critical Care Medicine Baltimore Maryland United States Division of Pulmonary and Critical Care Medicine, School of Medicine, Johns Hopkins University - Baltimore, Maryland, United States.; 2 Boston University Chobanian & Avedisian School of Medicine Boston Massachusetts United States Boston University Chobanian & Avedisian School of Medicine - Boston, Massachusetts, United States.; 3 Johns Hopkins University School of Medicine Division of Neonatology and General Internal Medicine Baltimore Maryland United States Division of Neonatology and General Internal Medicine, School of Medicine, Johns Hopkins University - Baltimore, Maryland, United States.; 4 Johns Hopkins University Bloomberg School of Public Health Baltimore Maryland United States Bloomberg School of Public Health, Johns Hopkins University - Baltimore, Maryland, United States.

## INTRODUCTION

Sepsis caused by fungal organisms is associated with higher mortality and longer time to diagnosis.^(1,2)^ Estimates of the incidence of fungal infections among patients with sepsis in the United States have not been reported since 2000 and did not specifically address those with septic shock.^(3)^ There have since been changes in sepsis management and available antifungal therapies. The main objective of this study is, among patients with new septic shock, to describe the incidence of diagnosed fungal infections and patterns of intravenous antifungal use in relation to the start of intravenous vasopressors.

## METHODS

We abstracted data from the electronic health record of five hospitals and restricted our cohort to adults with sepsis admitted between January 2017 and June 2024 by applying Angus sepsis algorithms (explicit sepsis diagnosis or combination of code for infection and a code for organ dysfunction) to ICD-10 codes labelled,^(4,5)^ as present on admission. To restrict the cohort to septic shock encounters, we only included encounters in which a vasopressor was administered. Diagnosed fungal infections were measured based on ICD-10 codes abstracted by trained billing coders and verified by attending physicians (Table 1S - Supplementary Material). Intravenous antifungal receipt was assessed 7 days before or after initiating vasopressors. Additional methodological details are available in the Supplementary Material.

## RESULTS

Among 23,232 hospitalizations for septic shock, 1,082 (4.7%) were coded as having a fungal infection (Table 2S - Supplementary Material). The most commonly implicated fungal pathogens were *Candida* (598/1,082, 55.3%) and *Aspergillus* (278/1,082, 25.7%; Table 1, Table 3S and Figure 1S [Supplementary Material]).

**Table 1:** Hospitalization characteristics according to presence of fungal diagnostic code among patients admitted with septic shock

Characteristic		Fungal infection code present	Fungal infection code absent	Total	p value
Encounters		1,082 (4.7)	22,150 (95.3)	23,232 (100.0)	
Mean age (years)		60.5 ± 15.4	64.6 ± 16.4	64.4 ± 16.4)	< 0.001
Female sex		477 (44.1)	10,431 (47.1)	10,908 (47.0)	0.053
Mean length of stay (days)		40.7 ± 45.3	19.6 ± 22.9	20.6 ± 24.8	< 0.001
Received IV antifungal within 7 days of vasopressor		551 (50.9)	2,861 (12.9)	3,412 (14.7)	< 0.001
	Micafungin*	333 (60.4)	1,675 (58.5)	2,008 (58.9)	0.409
	Fluconazole	84 (15.2)	882 (30.8)	966 (28.3)	< 0.001
	Other†	134 (24.3)	304 (10.6)	438 (12.8)	< 0.001
Immunocompromised‡		822 (76.0)	14,685 (66.3)	15,507 (66.7)	< 0.001
	Diabetes with end-organ damage	342 (31.6)	7,742 (35.0)	8,084 (34.8)	0.024
	Malignancy	439 (40.6)	6,987 (31.5)	7,426 (32.0)	< 0.001
	Chronic kidney disease	290 (26.8)	4,384 (19.8)	4,674 (20.1)	< 0.001
	Liver disease with cirrhosis	180 (16.6)	2,271 (10.3)	2,451 (10.6)	< 0.001
	HIV/AIDS	13 (1.2)	137 (0.6)	150 (0.6)	0.019
In-hospital death		398 (36.8)	6,642 (30.0)	7,040 (30.3)	< 0.001
Fungal genus§					
	*Candida*	598 (55.3)	n.d.	n.d.	
	*Aspergillus*	278 (25.7)	n.d.	n.d.	
	Other/unspecified¶	163 (15.1)	n.d.	n.d.	
	*Histoplasmosis*	53 (4.9)	n.d.	n.d.	
	*Cryptococcus*	32 (3.0)	n.d.	n.d.	
	*Mucor*	20 (1.8)	n.d.	n.d.	
	*Coccidioides*	2 (0.2)	n.d.	n.d.	

n.d. - not determined. p values generated using two-sided Student's t-test for continuous and Chi-squared test for categorical measures.

* Percentages calculated using number of patients who received any antifungal medication;

† includes amphotericin, caspofungin, isavuconazole, and voriconazole;

‡ totals exceed 100% due to patients with ≥ 1 condition;

§ percentages are of the 1,082 encounters with fungal diagnosis code. Totals exceed 100% due to encounters with ≥ 2 diagnosis codes;

¶ includes diagnosis codes B48.8 (other specified mycoses) and B49 (unspecified mycosis). Results expressed as n (%) or mean ± standard deviation. Additional information is in tables 2S and 3S (Supplementary Material).

Compared to those without, patients with coded fungal diagnoses were younger (60.5 *versus* 64.6 years; p < 0.001) with a higher prevalence of immunocompromising comorbidities (76.0% *versus* 66.3%; p < 0.001); they experienced longer hospitalizations (40.7 *versus* 19.6 days; p < 0.001) and more frequent in-hospital death (36.8% *versus* 30.0%; p < 0.001).

Overall, 14.7% (3,412/23,232) of patients with septic shock received an intravenous antifungal within 7 days of vasopressor initiation; of these, 16.1% (551/3,412) were ultimately diagnosed with (and received an ICD-10 code for) a fungal infection, and 83.9% (2,861/3,412) were not. Septic shock patients with a fungal diagnosis code were more likely to receive an intravenous antifungal compared to those without (50.9% *versus* 12.9%; p < 0.001). Micafungin was the most common (2,008/3,412; 58.9%), followed by fluconazole (966/3,412; 28.3%).

Of 3,412 septic shock patients who received an antifungal within 7 days of a vasopressor, 21.3% (727/3,412) were started on the antifungal medication before or concurrent with the vasopressor, and 82.9% by 72 hours later (Figure 1). The average time of antifungal initiation was 22.6 hours after the initiation of vasopressors, and did not differ significantly according to the presence of a fungal diagnosis code (p = 0.317).

**Figure 1:**
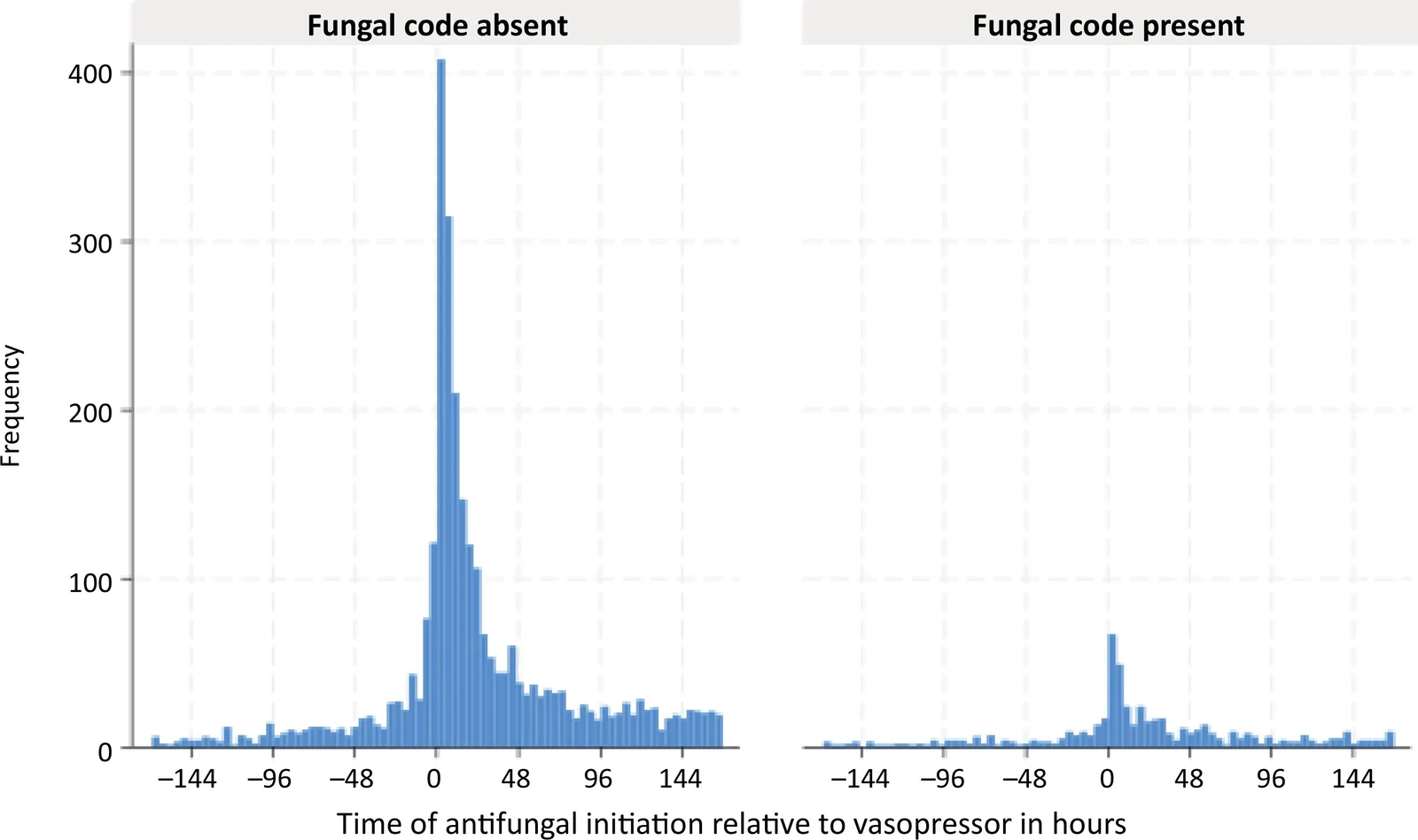
Time of antifungal administration relative to vasopressors according to whether or not a fungal diagnostic code was given.

Positive values represent antifungals administered after vasopressors, and negative values represent antifungals administered before vasopressors.

## DISCUSSION

In this hospital system, most antifungal use (83.9%) was in patients without a fungal diagnosis code, and one in eight septic shock patients without a fungal diagnosis received an intravenous antifungal. The unadjusted incidence of fungal diagnostic codes was higher than previous estimates (47 *versus* 3.5 - 16.5 per 1,000), possibly because we focused on United States septic shock encounters whereas these European estimates were derived from a broader sample of all intensive care unit patients.^(6,7)^

It is important to note that our study relies on diagnosis codes based on bedside clinician documentation of their judgment to define fungal infections, which is different than retrospective adjudicated research review.^(8,9)^

Only 16.1% of patients treated with intravenous antifungals were ultimately diagnosed with a fungal infection. A validated approach to risk stratification might improve antifungal use, safety, stewardship, and outcomes among patients with septic shock.

## Data Availability

Data cannot be made publicly available beause contain protected health information.
