# Supplementary material for: Antifungal use with and without fungal diagnoses among patients with septic shock in a retrospective multicenter cohort
Source: Crit Care Sci. 2026 Aug 25;38:e20260094. doi: 10.62675/2965-2774.20260094 (PMC13690028; doi:10.62675/2965-2774.20260094)
Supplement: Supplementary file 1 [file 2965-2774-ccsci-38-e20260094-Suppl01.pdf]

Critical Care Science
https://doi.org/10.62675/2965-2774.20260094

SUPPLEMENTARY MATERIAL

Antifungal use with and without fungal diagnoses among patients with septic shock in a retrospective multicenter cohort

Robert J. Flick[1], Li Yan[1], Nicholas A. Bosch[2], Anica C. Law[2], Khyzer B. Aziz[3], Chad H. Hochberg[1], Joseph Levy[4], Theodore J. Iwashyna[1]

SUPPLEMENTAL METHODOLOGY DETAILS

Study setting

Five hospitals in Maryland and Washington, D.C. were included, representing all hospitals that care for adults in the Johns Hopkins Health System: Johns Hopkins Hospital, Bayview Medical Center, Sibley Memorial Hospital, and Suburban Hospital.

Definition of septic shock

We required encounters to meet two criteria to be classified as septic shock, following the methods of Bosch et al.[(1)] First, a vasopressor must have been administered. This was abstracted from the medication administration record (MAR) in the electronic health record. We defined a vasopressor as any of the following: vasopressin, norepinephrine, phenylephrine, epinephrine, and dopamine. Second, the encounter must have carried ICD-10 diagnostic codes explicit for severe sepsis, septic shock (R65.20 or R65.21), or for a specific infection.

Definition of fungal infection

A subset of encounters was classified as having a fungal infection that could plausibly contribute to the development of septic shock. Two practicing clinician authors reviewed the ICD-10 and included all fungal infections that could reasonably be expected to cause septic shock (Table 1S). Encounters were classified as having a fungal diagnostic code if they carried one of these codes. When a diagnostic code included the specific fungal organism, we used this information to calculate specific pathogen sums.

Measurement of antifungal medication administration

We included all available intravenous antifungal medications: amphotericin B (all formulations), anidulafungin, caspofungin, fluconazole, isavuconazole, micafungin, posaconazole, rezafungin, and voriconazole. We reviewed the National Drug Code and extracted all possible naming variations for these medications. We defined encounters with antifungal use as those in which any of the aforementioned were administered intravenously, according to the MAR, within 7 days before or after the initiation of the first vasopressor medication. If an intravenous antifungal medication was administered only outside of the 7-day window, then the encounter was classified as no antifungal use.

Statistical testing

Continuous variables were compared using unpaired two-tailed Student's t-tests. Categorical variables were compared using Chi-squared tests. Rates of fungal diagnoses and antifungal use were annualized, and unadjusted linear regression was used to assess trends. We considered a p value less than 0.05 statistically significant.

This is an open access article under the CC BY license (https://creativecommons.org/licenses/by/4.0/).

Table 1S - ICD-10 codes used to define presence of high-risk fungal infection

Fungal infection	ICD-10 codes
Aspergillosis	B44.0, B44.1, B44.7, B44.9
Blastomycosis	B40.1-B40.7, B40.81, B40.89, B40.9
Candidiasis	B37.6, B37.7, B37.9
Coccidioidomycosis	B38.0-B38.4, B38.7, B38.89, B38.9
Cryptococcosis	B45.0-B45.9
Histoplasmosis	B39.0-B39.9
Other specified mycoses	B48.8
Paracoccidioidomycosis	B41.0-B41.9
Penicillosis	B48.4
Unspecified mycosis	B49
Zygomycosis	B46.0-B46.9

Table 2S - Characteristics of septic shock hospitalizations according to presence of fungal infection

	Fungal infection coded	Fungal infection not coded	All	p value
Total encounters	1,082 (4.7)	22,150 (95.3)	23,232 (100)	
Hospital				
Johns Hopkins Hospital	764 (70.6)	10,907 (49.2)	11,671 (50.2)	< 0.001
Bayview Medical Center	143 (13.2)	4,790 (21.6)	4,933 (21.2)	
Howard County General	77 (7.1)	2,816 (12.7)	2,893 (12.5)	
Suburban Hospital	55 (5.1)	2,459 (11.1)	2,514 (10.8)	
Sibley Memorial Hospital	43 (4.0)	1,178 (5.3)	1,221 (5.3)	
Mean age (years)	60.5 ± 15.4	64.6 ± 16.4	64.4 ± 16.4	< 0.001
Female sex	477 (44.1)	10,431 (47.1)	10,908 (47.0)	< 0.001
Received IV antifungal	551 (50.9)	2,861 (12.9)	3,412 (14.7)	< 0.001
Micafungin	333 (60.4)	1,675 (58.6)	2,008 (58.9)	
Fluconazole	84 (15.3)	882 (30.8)	966 (28.3)	
Voriconazole	51 (9.3)	90 (3.2)	141 (4.1)	
Amphotericin	45 (8.2)	85 (3.0)	130 (3.8)	
Isavuconazole	30 (5.4)	91 (3.2)	121 (3.6)	
Caspofungin	8 (1.5)	38 (1.3)	46 (1.4)	
Died or discharged to hospice	472 (43.6)	8,004 (36.1)	8,476 (36.5)	< 0.001
Mean length of stay (days)	40.7 ± 45.3	19.6 ± 22.9	20.6 ± 24.8	< 0.001
Highest SOFA score 24 hours after admission	5.7 (3.7)	5.9 (3.5)	5.9 (3.5)	.086
Highest SOFA score during admission	11.3 (3.8)	9.3 (3.6)	9.4 (3.6)	< 0.001
Elixhauser comorbidities	10.1 ± 4.1	9.1 ± 4.3	9.2 ± 4.3	< 0.001

SOFA - Sequential Organ Failure Assessment. Results expressed as n (%), mean ± standard deviation.

Table 3S - Clinical characteristics of 1,082 septic shock hospitalizations with a fungal infection according to vital status

	Died before discharge	Discharged alive	All	p value
Total encounters	398 (36.8)	684 (63.2)	1,082 (100)	
Received IV antifungal within 7 days of vasopressor	212 (53.2)	339 (49.6)	551 (50.9)	0.240
Pathogen				
Candida	204 (51.3)	394 (57.6)	598 (55.3)	0.043
Aspergillus	115 (28.9)	163 (23.8)	278 (25.7)	0.066
Unspecified	64 (16.1)	99 (14.5)	163 (15.1)	0.476
Histoplasmosis	20 (5.0)	33 (4.8)	53 (4.9)	0.883
Cryptococcus	16 (4.0)	16 (2.3)	32 (3.0)	0.116
Mucormycosis	10 (2.5)	10 (1.5)	20 (1.8)	0.216
Coccidiomycosis	1 (0.3)	1 (0.1)	2 (0.2)	0.698

Results expressed as n (%).

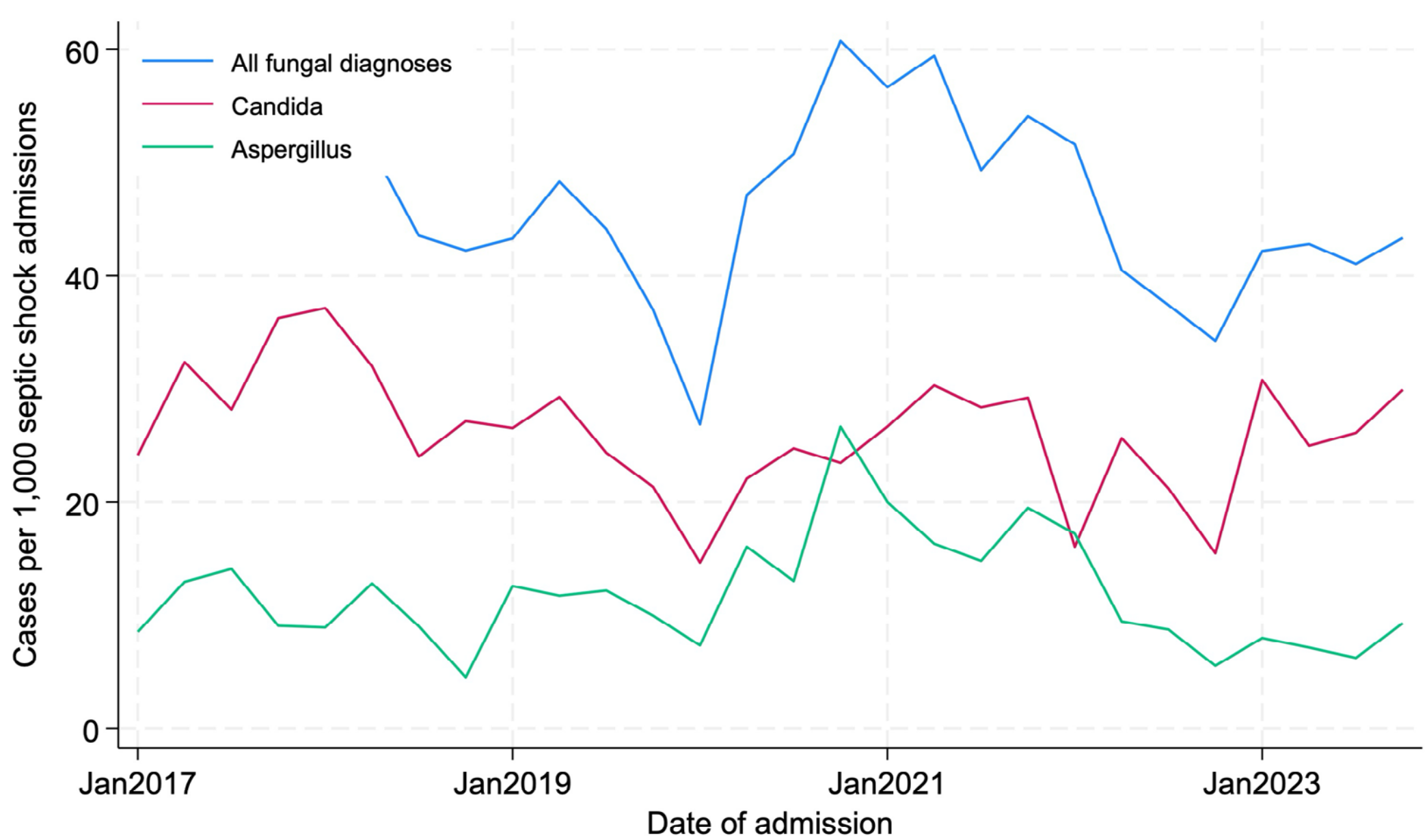

Figure 1S - Temporal trends in the incidence of fungal diagnoses per 1,000 septic shock admissions disaggregated by specific pathogen.

REFERENCE

1. Bosch NA, Law AC, Rucci JM, Peterson D, Walkey AJ. Predictive Validity of the Sequential Organ Failure Assessment Score versus Claims-based Scores among Critically Ill Patients. Ann Am Thorac Soc. 2022;19(6):1072-6.
